# Structure and function of aldopentose catabolism enzymes involved in oxidative non-phosphorylative pathways

**DOI:** 10.1186/s13068-022-02252-5

**Published:** 2022-12-28

**Authors:** Yaxin Ren, Veikko Eronen, Martina Blomster Andberg, Anu Koivula, Nina Hakulinen

**Affiliations:** 1 Department of Chemistry, University of Eastern Finland, 111, 80101 Joensuu, Finland; 2 VTT Technical Research Centre of Finland Ltd, Espoo, Finland

**Keywords:** Aldopentose, Non-phosphorylative pathways, Pentose catabolism, Aldose-1-dehydrogenase, Lactonase, Sugar acid dehydratase, Ketoglutarate-semialdehyde dehydrogenase, Aldolase

## Abstract

Platform chemicals and polymer precursors can be produced via enzymatic pathways starting from lignocellulosic waste materials. The hemicellulose fraction of lignocellulose contains aldopentose sugars, such as d-xylose and l-arabinose, which can be enzymatically converted into various biobased products by microbial non-phosphorylated oxidative pathways. The Weimberg and Dahms pathways convert pentose sugars into α-ketoglutarate, or pyruvate and glycolaldehyde, respectively, which then serve as precursors for further conversion into a wide range of industrial products. In this review, we summarize the known three-dimensional structures of the enzymes involved in oxidative non-phosphorylative pathways of pentose catabolism. Key structural features and reaction mechanisms of a diverse set of enzymes responsible for the catalytic steps in the reactions are analysed and discussed.

## Background

Excessive exploitation of fossil resources and severe deterioration of the natural environment have become serious challenges for further development of human society, and has led to intense exploration for alternative sources of energy, raw materials and food [1]. Lignocellulose offers an alternative raw material that is abundant, cheap and renewable [2]. In particular utilization of various types of lignocellulosic waste, such as from agriculture, forestry, agroindustry and municipalities, as feedstock can promote resource efficiency and circular economic goals without affecting the food supply [3]. Industrial biotechnology offers sustainable approaches to converting lignocellulosic wastes to fuels, chemicals and materials. To be economically feasible, all fractions of lignocellulosics (the major components being cellulose, hemicellulose and lignin) need, however, to be considered. Concerning sugars, the most common monosaccharide found in plant biomasses is d-glucose, a 6-carbon (C6) hexose sugar, which is a structural component of cellulose [4]. The second most abundant sugar is a 5-carbon (C5) pentose sugar, d-xylose, which is especially abundant in xylan-rich hemicelluloses [5, 6]. l-arabinose, another type of C5 pentose sugar, is found in plant polysaccharides, hemicelluloses and pectin.

**Fig. 1:**
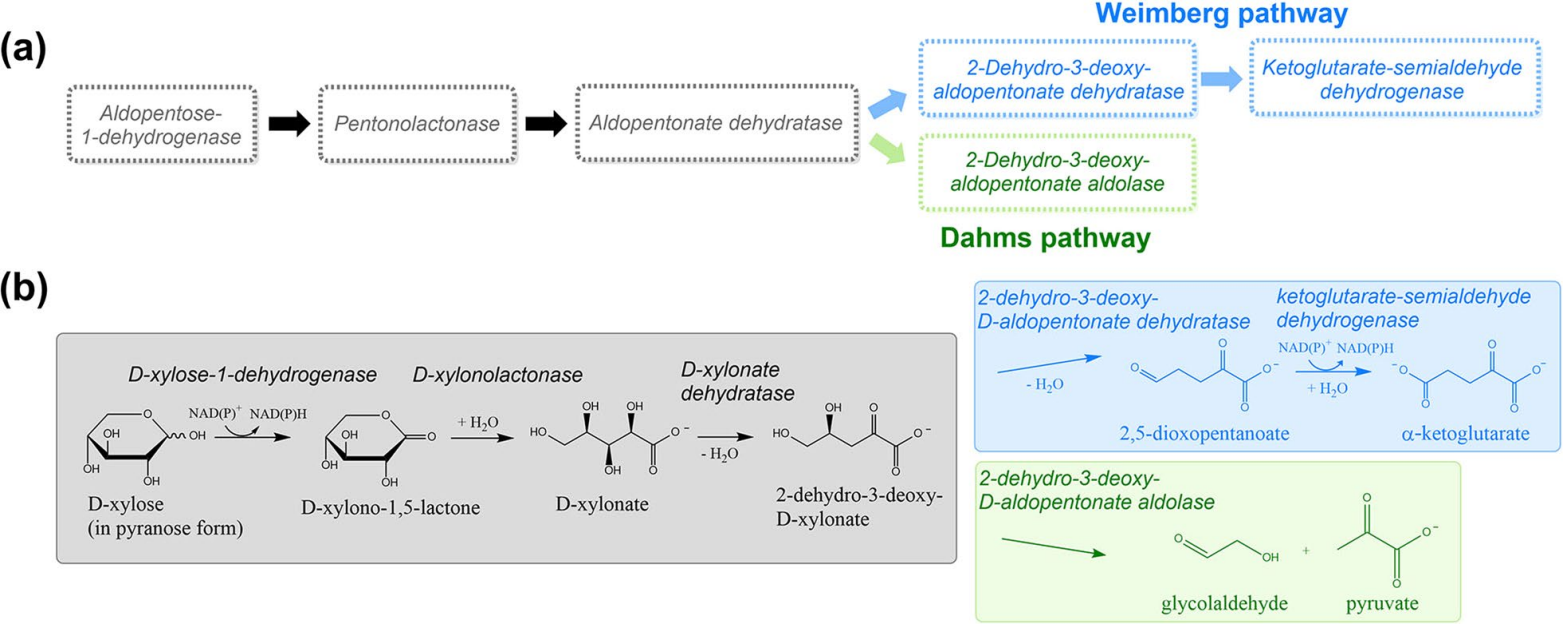
**a** General scheme of oxidative non-phosphorylative pathways for catabolism of pentose sugars. Weimberg and Dahms pathway enzymes are shown in blue and green, respectively, and the collective upstream steps in the pathways are shown in grey. **b** Enzymes involved in Weimberg/Dahms catabolism of d-xylose

Efficient biotechnological use of lignocellulosic waste will require microorganisms (such as bacteria or yeast) that possess metabolic pathways for utilizing biomass-derived monosaccharides. Currently, most biotechnological processes rely on d-glucose as the primary carbon source, but utilization of abundant biomass-derived pentose sugars is also of importance [7, 8]. Three main microbial pathways for pentose sugar catabolism, including d-xylose, l-arabinose and d-arabinose, have been described [9]. In the first pathway, mainly found in bacteria, the conversion uses isomerases, kinases and epimerases to produce d-xylulose-5-phosphate, which then can be further utilized by the pentose phosphate pathway [10]. In the second pathway, commonly found in yeast and fungi, pentoses are metabolized by reductases, dehydrogenases and kinases to also produce d-xylulose-5-phosphate [11]. The third pathway of pentose sugar catabolism, found in archaea and bacteria, is an oxidative, non-phosphorylative pathway, originally discovered by Weimberg in *Pseudomonas fragi* [12]*.* Here, pentose sugars are converted through 2-dehydro-3-deoxy-aldopentonate to α-ketoglutarate, which is an intermediate in the citric acid cycle (Fig. 1). In the oxidative Dahms pathway [13], a pentose sugar is converted through 2-dehydro-3-deoxy-aldopentonate to glycolaldehyde and pyruvate (Fig. 1). The homologous pathway of Dahms pathway is also described in the catabolism of deoxyhexoses, for example, l-rhamnose and l-fucose [14, 15]. Furthermore, Weimberg/Dahms pathways are analogous to the non-phosphorylative Entner–Doudoroff (ED) pathway for hexose sugars. These non-phosphorylative metabolic pathways have the potential for efficient production of high-value bioproducts as an alternative to more traditional metabolic pathways, which consist of more steps and have more complex regulation that limits production yields and rates. Lately, Watanabe et al. found a third non-phosphorylative route of pentose metabolism in *Herbaspirillum huttiense* IAM 15032 [16] by analysis of gene clustering, in which the intermediate 2-dehydro-3-deoxy-aldopentonate is converted to glycolate and pyruvate via 5-hydroxy-2,4-dioxo-pentanonate. The gene cluster analysis has an increasing role in the discovery of new routes and novel enzymes.

In the Weimberg pathway (Fig. 1), a pentose sugar is first oxidized to a lactone by an aldopentose-1-dehydrogenase enzyme, followed by the lactone ring being opened to a sugar acid by a pentonolactonase enzyme. Two sequential dehydration steps then follow: in the first step, an aldopentonate dehydratase converts the sugar acid to 2-dehydro-3-deoxy-aldopentonate, which is then converted to 2,5-dioxopentanoate by another dehydratase. In the final reaction step of the Weimberg pathway, 2,5-dioxopentanoate is converted to α-ketoglutarate by α-ketoglutarate-semialdehyde dehydrogenase enzyme. In the Dahms pathway, the 2-dehydro-3-deoxy-aldopentonate is directly split into pyruvate and glycolaldehyde by an aldolase enzyme, while the upstream steps in the pathway are in common with the Weimberg pathway.

Recent studies have increasingly focused on metabolic engineering of the Weimberg/Dahms pathways for production of biobased compounds, i.e., glycolic acid, glutaric acid, ethylene glycol, 1,2,4-butanetriol and 1,4-butanediol, which can be used in the textile, food, pharmaceutical and chemical industries, among others [3, 8, 17–20]. In this review, we provide an overview of the structural characteristics of Weimberg/Dahms pathway enzymes and describe what is known of their catalytic mechanisms and enzyme families.

## Aldopentose-1-dehydrogenases

The first step in the Weimberg/Dahms pathways is the oxidation of a pentose sugar to the corresponding lactone by aldopentose-1-dehydrogenases. In principle, l-arabinose is oxidized by l-arabinose dehydrogenase (E.C. 1.1.1.46 for the NAD- or 1.1.1.376 for the NAD/NADP-dependent forms), d-arabinose by d-arabinose dehydrogenase (E.C. 1.1.1.116 for the NAD- or 1.1.1.117 for the NADP-dependent forms) and d-xylose by d-xylose dehydrogenase (E.C. 1.1.1.175 for the NAD- and 1.1.1.179 for the NADP-dependent forms). However, many aldose-1-dehydrogenases have wide substrate specificity, and they can act on various aldopentoses as well as aldohexoses (E.C. 1.1.1.121). The general term aldose-1-dehydrogenase is used when enzymes utilize NAD or NADP as cofactors to oxidize aldoses to aldonolactones.

At present, there are only a few crystal structures of aldose-1-dehydrogenases with preference for pentose sugars available from the Protein Data Bank (PDB) (Table 1). These aldose-1-dehydrogenases belong to either the dehydrogenase/reductase superfamily or the Gfo/Idh/MocA superfamily.

**Table 1:** Available crystal structures of aldose-1-dehydrogenases acting on pentose sugars

Enzyme (Short name in this article) EC number	PDB code	Organism	Resolution (Å)	Ligands	Quaternary structure	Refs.
Dehydrogenase/reductase superfamily: MDR family						
d-arabinose dehydrogenase (*Ss* ADH) EC 1.1.1.117	2H6E	*Saccharolobus solfataricus*	1.80	Zn^2+^	Tetramer	[21]
d-glucose dehydrogenase (*Ss* GDH) EC 1.1.1.119	2CD9 2CDA 2CDB 2CDC	*Saccharolobus solfataricus*	1.80 2.28 1.60 1.5	Zn^2+^ Zn^2+^, NADP Zn^2+^, NADP, d-glucose Zn^2+^, NADP, d-xylose	Tetramer	[22]
Gfo/Idh/MocA superfamily						
l-arabinose dehydrogenase (*Ab* ADH) EC 1.1.1.376	6JNJ 6JNK 7CGQ	*Azospirillum brasiliensis*	1.50 2.20 2.21	– NADP NADP, L-arabinose	Dimer	[23, 24]
d-galactose-1-dehydrogenase (*Re* GDH) EC 1.1.1.120	4EW6	*Rhizobium etli*	2.30	–	Dimer	[25]
Aldose–aldose oxidoreductase EC 1.1.99.-	5A02 5A03 5A04 5A05 5A06	*Caulobacter crescentus*	2.00 1.85 1.70 1.90 1.84	NADP, glycerol NADP, d-xylose NADP, d-glucose NADP, maltotriose NADP, d-sorbitol	Dimer	[26]

### Dehydrogenase/reductase superfamily

The dehydrogenase/reductase superfamily is a very large and versatile superfamily of NAD(P)-dependent proteins with high sequence diversity and a wide range of catalytic activities [27]. The first solved protein structure of the family, described in 1976, was that from a horse liver alcohol dehydrogenase [28]. Based on polypeptide chain length, distinct sequence motifs, and structural comparisons, the dehydrogenase/reductase superfamily can be further divided into short-, medium- and long-chain dehydrogenase/reductase superfamilies (SDR, MDR and LDR, respectively) [29]. d-arabinose dehydrogenase from *S. solfataricus* [30] as well as l-arabinose dehydrogenase from the bacterial species *R. leguminosarum* [31], and a d-xylose dehydrogenase from the archaean species *H. marismortui* [32],belong to the MDR superfamily. Conversely, d-xylose dehydrogenase of the bacterial species *C. crescentus* [33] and an l-arabinose dehydrogenase of the archaean species *H. volcanii* [34] are reported to belong to the SDR superfamily based on sequence comparisons. No X-ray structures of pentose dehydrogenases belonging to the SDR superfamily have been published to date. However, there are some structures (unbound and ligand bound) of a deoxyhexose dehydrogenase of SDR family, the l-rhamnose-1-dehydrogenase from *Azotobacter vinelandii* [35]. SDR- and MDR-family pentose dehydrogenases belong to a branch of alcohol/polyol/sugar dehydrogenases along with the alcohol dehydrogenases, sorbitol dehydrogenases and aldohexose dehydrogenases [36].

A crystal structure of the d-arabinose dehydrogenase from *S. solfataricus* (hereafter referred to as *Ss* ADH; PDB code 2H6E) was determined in 2007 [21]. The year before, the X-ray structure of a glucose dehydrogenase, also from *S. solfataricus* (hereafter referred to as *Ss* GDH), had been solved in apo and complex forms (PDB codes 2CD9, 2CDA, 2CDB, and 2CDC). *Ss* GDH catalyzes the oxidation of glucose to gluconate in the non-phosphorylated ED pathway, but it is also able to act on d-galactose, d-xylose and l-arabinose [37]. *Ss* GDH shares only 19% sequence identity with *Ss* ADH, but the enzymes are structurally and functionally very similar.

**Fig. 2:**
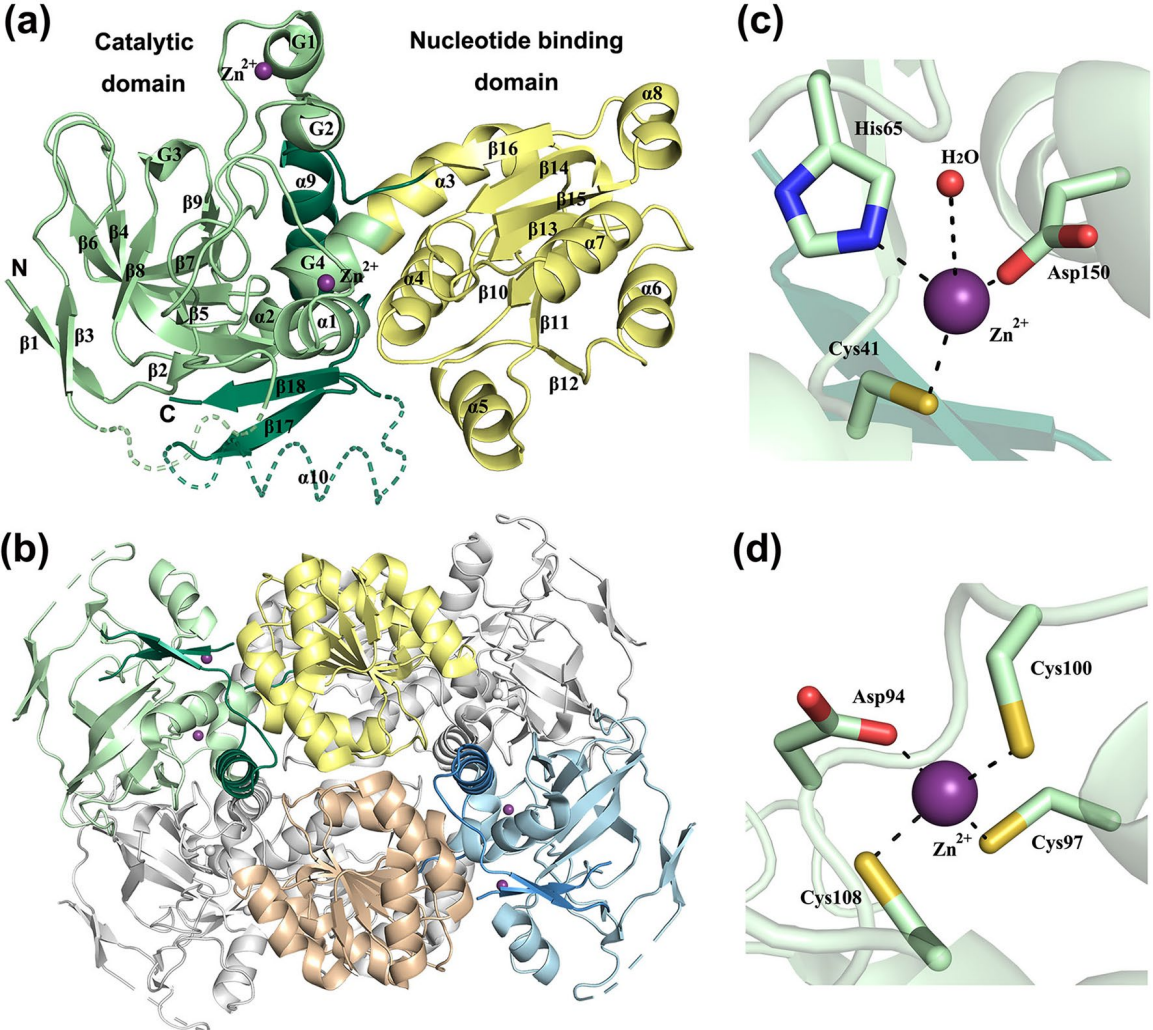
Crystal structure of d-arabinose dehydrogenase from *S. solfataricus* (PDB: 2H6E). **a** Overall fold is represented by a ribbon diagram. The catalytic domain is shown in green, where the N-terminal and C-terminal segments of the catalytic domain are shown in light and dark green, respectively. The nucleotide binding domain is shown in yellow. Zn^2+^ ion is shown as a purple sphere. The dashed line represents a missing part of the crystal structure due to weak electron density (here reconstructed by homology modelling). **b** Tetrameric quaternary structure generated by crystallographic symmetry operations. **c** Coordination geometry of the catalytic Zn^2+^ ion. **d** Coordination geometry of the structural Zn.^2+^ ion. (All structural figures in this article were prepared using Pymol [40] software)

D-arabinose dehydrogenase *Ss* ADH consists of 344 amino acids, and it folds into a catalytic domain (residues 1–154 and residues 292–344), and a nucleotide binding domain (residues 155–291) (Fig. 2a) [21]. MDR superfamily proteins typically have these two domains, where the N-terminal domain is responsible for substrate binding and the C-terminal domain with a Rossmann fold is responsible for nucleotide binding. The catalytic domain of *Ss* ADH has an N-terminal segment composed of two α-helices (α1, α2), four 3_10_-helices (G1–G4), and nine β-strands (β1–β9). The C-terminal segment is composed of α-helix α9 (and a predicted additional α10) along with two β-strands (β17, β18). The core of the catalytic domain of Ss ADH is formed by five antiparallel β-strands (β4, β6–β9) and two parallel β-strands (β17, β18). The C-terminal nucleotide binding domain of *Ss* ADH has a classical Rossmann fold comprised of a six-stranded parallel β-sheet (β10–β16) surrounded by six α-helices (α3–α8). The overall fold is very similar to that of *Ss* GDH and other MDR dehydrogenases. The quaternary structure of *Ss* ADH is a homotetramer (Fig. 2b), which is a common quaternary structure of MDR enzymes, but monomers, dimers and trimers have also been observed among MDR family members [38]. The structurally related glucose dehydrogenase *Ss* GDH is a homotetramer [22].

In the catalytic domain, *Ss* ADH has two Zn^2+^ ions, one being catalytic and the other structural. The catalytic Zn^2+^ ion is tetrahedrally bound by Cys41, His65, Asp150 and a water molecule (Fig. 2c). The Cys and His residues are well-conserved among MDR family members, but the third ligand can be a Glu or Cys residue. In *Ss* GDH, Cys69 and His66 residues, along with Glu67 from the β7-strand and a water molecule, are coordinated to the Zn^2+^ ion. In addition to the catalytic Zn^2+^ ion, there is the structural Zn^2+^ ion, which is bound to Asp94, Cys97, Cys100 and Cys108 in *Ss* ADH (Fig. 2d), similar to that in alcohol dehydrogenases. The structural Zn^2+^ ion stabilizes the loop, which protrudes from the catalytic domain. It has been observed that removal of the structural Zn^2+^ ion in yeast alcohol dehydrogenase results in decreased thermostability [39].

The active site of MDR proteins is located in a deep crevice between the two domains that accommodates the NAD(P) and substrate. At the moment, there is no available complex structure of *Ss* ADH or any other aldopentose-1-dehydrogenase of the dehydrogenase/reductase superfamily but the structure of *Ss* GDH complexed with d-glucose or d-xylose has been solved [22]. Based on structural similarities, *Ss* ADH most likely operates via the same reaction mechanism as that observed for *Ss* GDH. The aldose sugar in the pyranose form is oxidized to aldonolactone, when a hydride from C1 of aldose is transferred to C4 of the nicotinamide ring, which is facilitated by proton abstraction from the hydroxyl group at C1 of the sugar ring. Structures of *Ss* GDH complexed with d-glucose or d-xylose were observed in the chair conformation and found to exist in the β-anomer, which allows C1 of the pyranose ring to be close to C4 of nicotinamide ring. d-arabinose dehydrogenase converts sugars with opposite stereo configuration at C2 and C3 (2S,3R in d-arabinose) as compared to d-glucose dehydrogenase (2R, 3S in d-glucose, d-xylose and l-arabinose), and therefore, it has been proposed that the pyranose ring needs to be flipped by 180° in *Ss* ADH [21], but this proposal still needs to be confirmed by the structure of ADH complexed with d-arabinose. It is also unclear whether an aldose substrate interacts with the Zn^2+^ ion via coordinated water or whether the C1 hydroxyl group of the substrate can form a covalent bond with Zn^2+^, making it penta-coordinated.

### Gfo/Idh/MocA superfamily

Proteins of the Gfo/Idh/MocA superfamily are NAD(P)-dependent oxidoreductases that act on a diverse set of substrates. The family name refers to glucose–fructose oxidoreductases, inositol 2-dehydrogenases, and the rhizopine catabolism protein MocA, but it also includes a growing number of other oxidoreductases [41]. The 6-phosphate glucose dehydrogenase from *Leuconostoc mesenteroides* was the first solved protein structure of the Gfo/Idh/MocA superfamily [42].

In 2019, the crystal structure of an l-arabinose dehydrogenase from the bacterial species *A. brasiliensis* (hereafter referred to as *Ab* ADH) was solved with and without the cofactor NADP (PDB codes 6JNK and 6JNJ) [23]. Very recently, its structure in a complex with l-arabinose (PDB code 7CGQ) was also published [24]. In addition, the crystal structure of a d-galactose-1-dehydrogenase from the bacterial species *R. etli* (hereafter referred to as *Re* GDH; PDB code 4EW6), has been solved by the Structural Genomics Research Consortium [25]. *Re* GDH is thought to be involved in galactose catabolism, but interestingly the orthologous enzyme from *Rhizobium leguminosarum* bv. *trifolii* was named l-arabinose/d-galactose-1-dehydrogenase due to its high catalytic efficiency toward l-arabinose, d-galactose and d-fucose [31]. The sequence identity of *Re* GDH with l-arabinose/d-galactose-1-dehydrogenase from *R. leguminosarum* is as high as 96%. The sequence identity between *Ab* ADH and *Re* GDH is 57%. The Gfo/Idh/MocA superfamily also contains an aldose–aldose oxidoreductase from *C. crescentus* (*Cc* AAOR), which was discovered through its sequence homology to the xylose dehydrogenases [43]. *Cc* AAOR has been shown to catalyze the concomitant oxidation and reduction of d-xylose and other aldose monosaccharides to the corresponding aldonolactones or alditols, respectively. *Cc* AAOR contains a tightly bound NADP cofactor, which is regenerated in this oxidation–reduction cycle. Several crystal structures of *Cc* AAOR have been solved including those complexed with d-xylose and d-glucose [26]. However, the sequence identity between *Ab* ADH and *Cc* AAOR is only 30%. *Cc* AAOR has been involved in co-production of d-xylonate and xylitol from d-xylose in *Saccharomyces cerevisiae *[44].

**Fig. 3:**
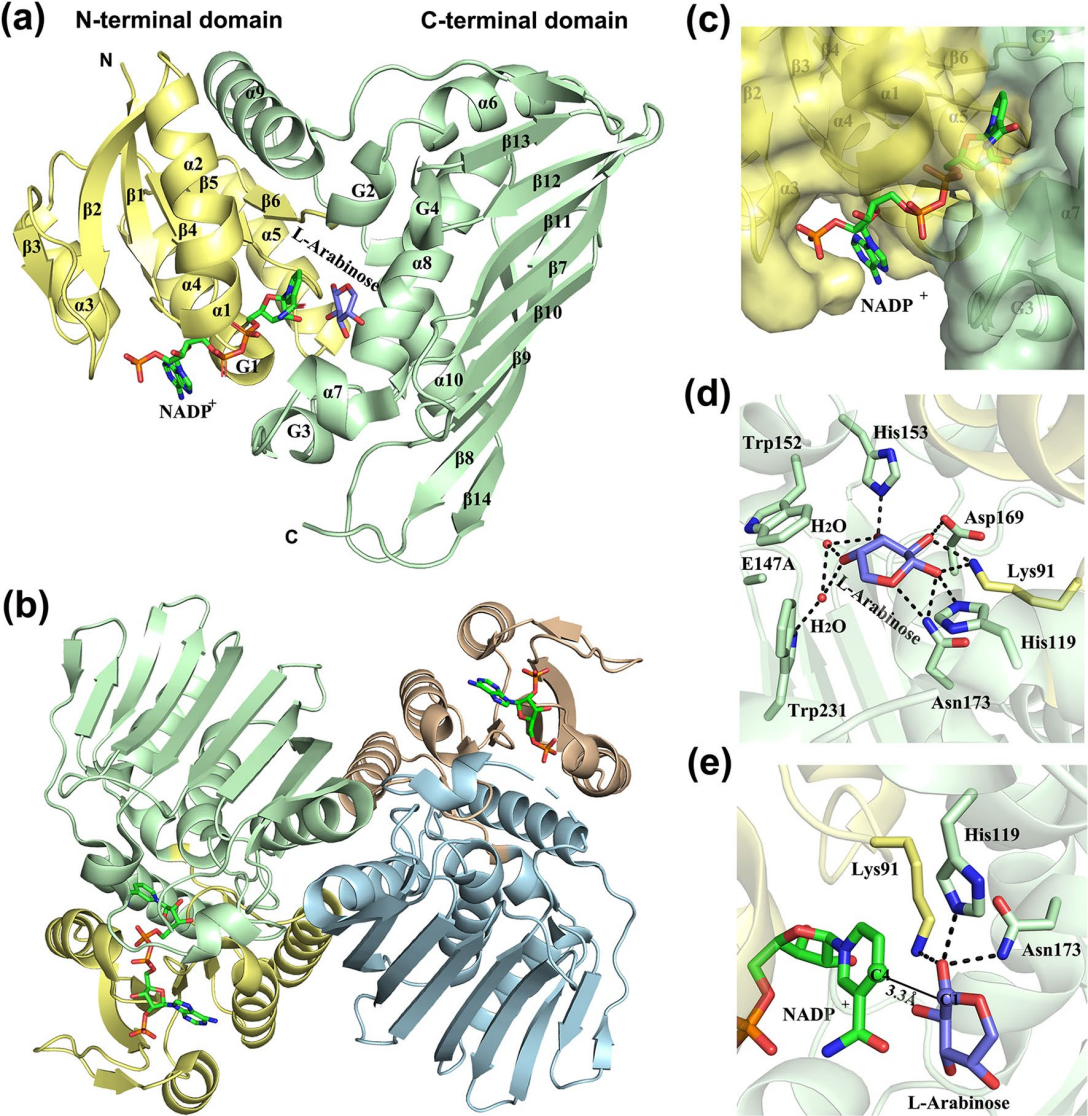
Crystal structure of L-arabinose dehydrogenase from *A. brasiliensis* (PDB: 7CGQ). **a** Overall fold is represented by a ribbon diagram. The N-terminal domain is shown in yellow, and the C-terminal domain in green. **b** Quaternary structure is a homodimer. **c** NADP-binding site is located in the crevice between the two domains. **d** Amino acid residues interacting with l-arabinose. **e** Hydrogen-bonding network around the O1 hydroxyl of l-arabinose. The distance from C1 of l-arabinose to C4 of the nucleotide is also shown

The L-arabinose dehydrogenase *Ab* ADH has the typical two-domain structure of Gfo/Idh/MocA family proteins (Fig. 3a). The N-terminal nucleotide-binding domain has a classical Rossmann fold with six-stranded parallel β-sheet (β1–β6) surrounded by five α-helices (kinked α1, α2, α3, α4 and α5) and one short 3_10_-helix G1. The C-terminal domain has a two-layer α/β-sandwich fold consisting of an eight-stranded β-sheet (β7–β14) and eight helices (five α-helices, α6–α10, and three 3_10_-helices, G2–G4). The C-terminal domain participates in oligomerization. Both *Ab* ADH and galactose dehydrogenase *Re* GDH shows structural similarity with *Cc* AAOR and glucose-6-phosphate dehydrogenases. *Ab* ADH and *Re* GDH exist as a homodimer, which is characteristic of Gfo/Idh/MocA proteins (Fig. 3b). Interestingly, *Cc* AAOR has a different dimerization mode compared with *Ab* ADH and *Re* GDH. In *Cc* AAOR, the β-sheets of two monomeric units are tightly packed together, and this dimerization creates an α–β–β–α sandwich-type structure at the dimeric interface.

The ternary complex of *Ab* ADH with L-arabinose and NADP shows that the active site is located in the crevice formed between the two domains (Fig. 3c) [24]. NADP is bound by three loops (β1–α1, β2–β3 and β4–α3) and one α-helix (α3) of the N-terminal domain. The region binding the 2′-phosphate is highly positively charged in *Ab* ADH, where in some NAD-preferred Gfo/Idh/MocA family proteins, this region contains hydrophobic or negatively charged residues [45–47]. l-arabinose is observed to interact through hydrogen bonding with Lys91, His119, Glu147, Trp152, His153, Asp169, Asn173 and Trp231 (Fig. 3d). The oxidation mechanism of Gfo/Idh/MocA proteins is known to be involved in the transfer of hydride from the C1 carbon of the substrate to the C4 carbon of the nicotinamide ring, coupled with deprotonation of the O1 hydroxyl of the substrate by a catalytic base. In general, a tyrosine or histidine residue functions as the catalytic base. In *Ab* ADH, three residues Lys91, His119 and Asn173, are located close to the C1 hydroxyl group of l-arabinose (Fig. 3e). Site-directed mutagenesis studies of *Ab* ADH showed that the H119N mutant retained its activity, but the activity of the N173Y and N173H mutants were reduced by three orders of magnitude compared to the wild type [24]. These results suggested that Asn173 in *Ab* ADH plays an important role in catalysis.

## Pentonolactonases

In the second step of the Weinberg/Dahms pathway, pentonolactone is hydrolyzed to pentoic acid. d-xylonolactone is catalyzed by xylonolactonase (E.C. 3.1.1.68), and l-arabinolactone by arabinolactonase (E.C. 3.1.1.15). This intramolecular ester bond hydrolysis step proceeds spontaneously but slowly at ambient temperature in vitro, although spontaneous lactone hydrolysis might proceed over a more reasonable timescale at elevated temperatures. The hyperthermophilic *Sulfolobus solfataricus,* for example, lacks an aldonolactonase gene in the pentose degradation gene cluster [30]. However, two genes encoding putative lactonases have been identified elsewhere in the *S. solfataricus* genome, but the involvement of these genes in pentose degradation is not yet clear. Structural information of pentonolactones is very limited and only very recently have crystal structures of xylonolactonase from *C. crescentus* complexed with d-xylose and 4-hydroxy-2-pyrrolidinone become available (PDB codes 7PLB, 7PLC and 7PLD) [48]. This enzyme, which is found in *C. crescentus* in the same operon as in the genes for the *Cc* XylB dehydrogenase and *Cc* XylD dehydratase enzymes, has been shown to improve formation of d-xylonic acid in in vitro enzyme cascade studies [49].

In addition to *C. crescentus* xylonolactonase [33], pentonolactonases have been characterized from *Azospirillum brasilense *[53] and *Haloferax volcanii *[54]*.* Based on sequence homology, pentonolactonases belong to the senescence marker protein (SMP30) superfamily, along with gluconolactonases, paraoxonases and luciferase regenerating enzymes. Available crystal structures of aldonolactonases belonging to the SMP30 superfamily are shown in Table 2. Gluconolactonase catalyses gluconolactone to gluconic acid [50], and SMP30 protein is reported to convert l-gulonate to l-gulonolactone in the vitamin C biosynthetic pathway, but also possess gluconolactonase activity [55]. In addition, SMP30 is sometimes called regucalcin due to its putative role in calcium homeostasis [56].

**Table 2:** Available crystal structures of aldonolactonases belonging to the SMP30 superfamily

Enzyme EC number	PDB code	Organism	Resolution (Å)	Ligands	Quaternary structure	Refs.
Gluconolactonase EC 3.1.1.17	3DR2	*Xanthomonas campestris*	1.67	Ca^2+^	Dimer	[50]
SMP30	3G4E 3G4H 4GNB 4GNC	*Homo sapiens*	1.42 1.92 1.50 1.75	Ca^2+^ Zn^2+^ Ca^2+^ Ca^2+^, anhydroglucitol	Monomer	[51]
SMP30	4GN7 4GN8 4GN9 4GNA	*Mus musculus*	1.95 1.70 2.00 1.85	Ca^2+^ Ca^2+^, anhydroglucitol Ca^2+^, D-glucose Ca^2+^, xylitol	Monomer	[52]
Xylonolactonase EC 3.1.1.68	7PLB 7PLC 7PLD	*Caulobacter crescentus*	1.73 2.15 1.70	Fe^2+^, D-xylose Fe^2+^, D-xylose Fe^2+^, 4-hydroxy-2-pyrrolidone	Monomer	[48]

**Fig. 4:**
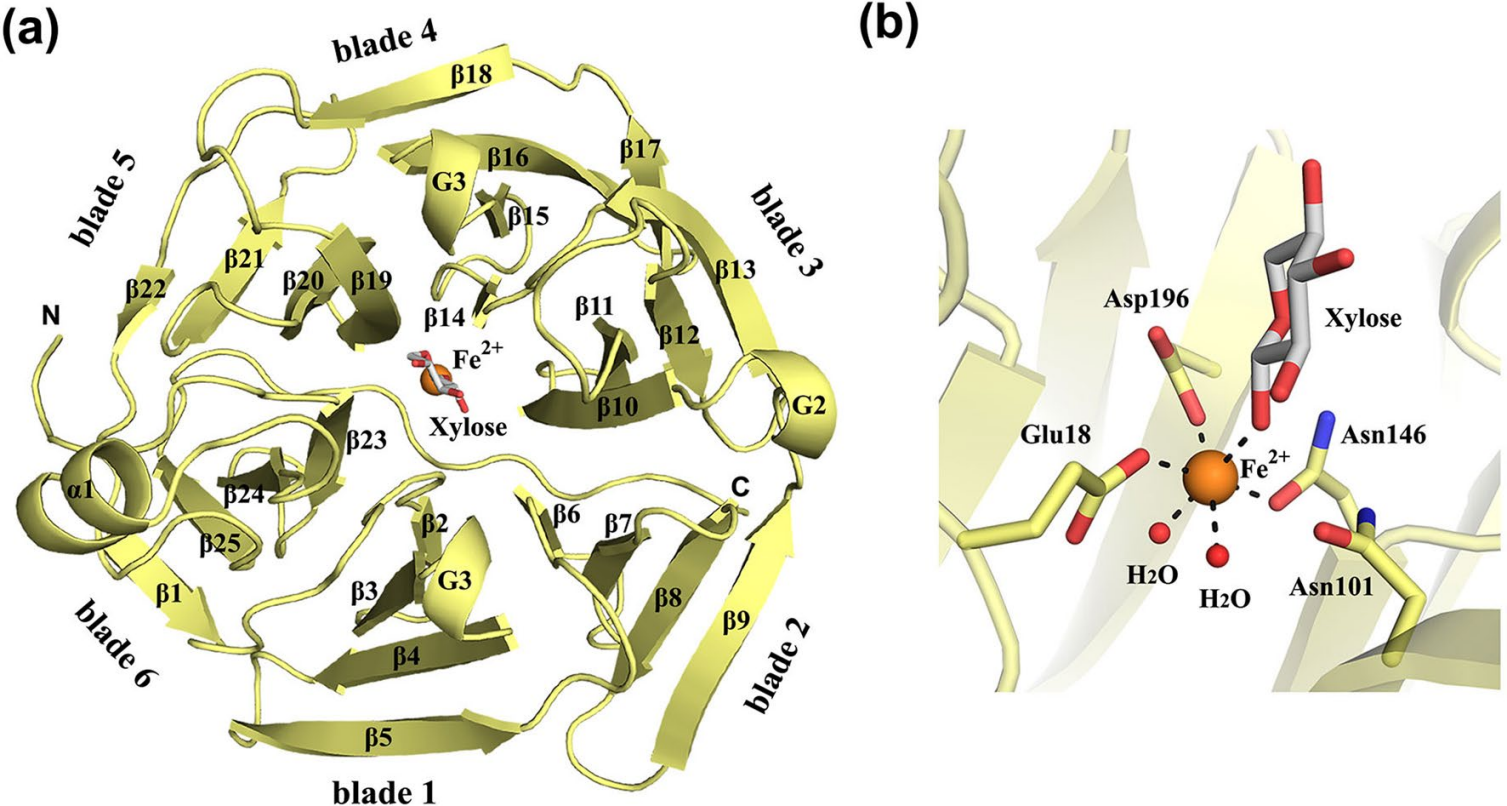
Crystal structure of xylonolactonase from *C. crescentus* (PDB: 7PLB). **a** Overall fold is represented by a ribbon diagram. The Fe^2+^ ion is indicated by an orange sphere. **b** Coordination geometry of the Fe^2+^ ion

Xylonolactonase is folded into a beta-propeller consisting of six blades, each of which is formed by four beta-strands (Fig. 4a). The active site of the enzyme is located in the central cavity, where the divalent metal binding site exists at the bottom of the cavity. In the solved *X. campestris* gluconolactonase structure, the bound metal ion at the catalytic site was determined to be Ca^2+^ [50], but many studies have reported that Zn^2+^ would be responsible for gluconolactonase activity [51]. On the other hand, a very recent study of *C. crescentus* xylonolactonase shows that the enzyme binds only Fe^2+^ ion with high specificity and affinity, and the other divalent metal cations are suggested to assist non-enzymatic hydrolysis by stabilizing the short-lived bicyclic intermediate during isomerization of lactone [57]. The Fe^2+^ ion, located in the active site of the enzyme, is bound by three conserved residues: Glu18, Asn146 and Asp196 (Fig. 4b), but not by the highly conserved Asn101. Xylose is also coordinated to Fe^2+^ together with two water molecules, which complete the octahedral coordination.

The site of substrate entrance into the SMP30 lactonases is located at the top of the metal-binding site. Although the SMP30 proteins have a lid-like structure that partly covers the substrate entrance [51], no such lid exists in *C. crescentus* xylonolactonase [46] or *X.* *campestris* gluconolactonase [49]. The crystal structures of *C.* *crescentus* xylonolactonase complexed with d-xylose or the substrate analogue 4-hydroxy-2-pyrrolidinone reveal the binding mode of the substrates [48]. d-xylose was observed in xylopyranose form and the equatorial O1 was bound to the Fe^2+^ ion over a short distance of 2.0 Å. Similarly, the carbonyl oxygen of 4-hydroxy-2-pyrrolidinone was 1.9 Å away from the Fe^2+^ ion. The crystal structures of mouse SMP30 protein complexed with the substrate analogue xylitol (PDB code 4GNA) or the product analogues 1, 5-anhydro-d-glucitol (PDB code 4GN8) and d-glucose (PDB code 4GN9) are also available [52]. The divalent metal ion and polar residues in the active site pocket interact with hydroxyl groups of the substrates.

The quaternary structures of the published aldonolactonases are either monomeric or dimeric. *C. crescentus* xylonolactonase, human and mouse SMP30 proteins are reported to be monomers, but gluconolactonase from *X. campestris* has been shown to exist as a disulphide-bonded homodimer. One structural Ca^2+^ ion was found to be at the interface of the monomeric subunits in the dimer and was suggested to stabilize the dimeric form.

## Aldopentonate dehydratases

In the third step of the Weimberg/Dahms pathways, an aldopentonate is converted to 2-dehydro-3-deoxy-aldopentonate by aldopentonate dehydratase. d-xylonate dehydratase (EC 4.2.1.82) converts d-xylonate to 2-dehydro-3-deoxy-d-xylonate, and l-arabinonate dehydratase (EC 4.2.1.25) converts l-arabinonate to 2-dehydro-3-deoxy-l-arabinonate by removing one water molecule. Currently, crystal structures of 2 homologous aldopentonate dehydratases, *R. leguminosarum* l-arabinonate dehydratase (hereafter referred to as *Rl* ADHT; (PDB codes 5J83, 5J84, 5J85) [58] and *C. crescentus* d-xylonate dehydratase (hereafter referred to as *Cc* XDHT; PDB code 5OYN) [59], have been solved. The 2 bacterial aldopentonate dehydratases have been shown to accept both pentonate and hexonate sugar acids as their substrates, being strictly stereospecific for the configuration of OH groups at C2 and C3 [60]. *Rl* ADHT is reported to have the highest catalytic efficiency (*k*_*cat*_*/K*_*m*_) for d-fuconate, followed by l-arabinonate and d-galactonate, while *Cc* XDHT prefers d-xylonate and d-gluconate. Metabolic engineering studies and in vitro enzyme cascade studies have indicated that the dehydratase reaction catalyzed by the *Cc* XDHT enzyme, which is a tetramer requiring a [2Fe–2S] cluster and Mg^2+^ ion for its activity [59–61], is a rate-limiting step in the Weimberg/Dahms pathways.

*Rl* ADHT and *Cc* XDHT enzymes belong to the iron–sulfur cluster-containing IlvD/EDD protein family, like many other aldopentonate dehydratases from bacterial species, such as *A. brasiliense* l-arabinonate dehydratase [53], *E.coli* D-xylonate dehydratase [67] and *Pseudomonas putida* d-xylonate dehydratase [68]. By contrast, aldopentonate dehydratases of the archaean species, such as d-arabinonate dehydratase from *Sulfolobus solfataricus* [30] and d-xylonate dehydratase from *Haloferax volcanii* [69]*,* are typically reported to belong to the enolase superfamily, but no crystal structure of any aldopentonate dehydratase belonging to the enolase family is currently known. This may in part be due to difficulties in expressing the archaeal aldopentonate dehydratases in heterologous hosts like *E.coli* [49]*.*

### IlvD/EDD superfamily

The IlvD/EDD protein family consists of various dehydratases that are either involved in short-chain amino acid biosynthesis (IlvD refers to isoleucine/leucine/valine dehydrates), or in carbohydrate metabolic pathways (EDD refers to Entner–Doudoroff dehydratases). All these enzymes are thought to have an iron–sulfur cluster at their active site. The [Fe–S] clusters are evolutionarily ancient prosthetic groups found in many metabolically important enzymes, and are suggested to participate in electron transfer and iron–sulphur storage, catalysis, regulation of gene expression, and in oligomer formation [70].

The first crystal structure of the IlvD/EDD family was an apo-form of 6-phosphogluconate dehydratase from *S. oneidensis* (PDB code 2GP4) solved by the Structural Genomics Consortium Project [62]. The enzyme 6-phosphogluconate dehydratase is involved in the classical ED pathway of glucose. The first aldopentonate dehydratase crystal structure became available in 2017 when the apo-, holo- and variant S480A structures of Rl ADHT (PDB codes 5J83, 5J84, 5J85, respectively) were solved [58]. A year later, the crystal structure of *Cc* XDHT (PDB code 5OYN) was also published [59]. Recently, holo-forms of dihydroxy acid dehydratase from *A. thaliana* (PDB codes 5YM0 and 5ZE4) [63], *M. tuberculosis* (PDB code 6OVT) [65] and *Synechocystis sp.* (PDB code 6NTE) [66] were determined (Table 3).

**Table 3:** Available crystal structures of IlvD/EDD enzymes

Enzyme (Short name in this article) EC number	PDB code	Organism	Resolution (Å)	Ligands	Quaternary structure	Refs.
6-Phosphogluconate dehydratase EC 4.2.1.12	2GP4	*Shewanella oneidensis*	2.49	–	Dimer	[62]
L-arabinonate dehydratase (*Rl* ADHT) EC 4.2.1.25	5J83 5J84 5J85	*Rhizobium leguminosarum* bv. *trifolii*	3.00 2.40 2.60	– [2Fe–2S], Mg^2+^ [2Fe–2S], Mg^2+^	Tetramer	[58]
d-xylonate dehydratase (*Cc* XDHT) EC 4.2.1.82	5OYN	*Caulobacter crescentus*	2.70	[2Fe–2S], Mg^2+^	Tetramer	[59]
Dihydroxyacid dehydratase E.C.4.2.1.9	5YM0 5ZE4	*Arabidopsis thaliana*	1.84 2.11	– [2Fe–2S], Mg^2+^	Dimer	[63, 64]
Dihydroxyacid dehydratase E.C.4.2.1.9	6OVT	*Mycobacterium tuberculosis*	1.88	[2Fe–2S], Mg^2+^	Tetramer	[65]
Dihydroxyacid dehydratase E.C.4.2.1.9	6NTE	*Synechocystis sp.*	2.33	–	Dimer	[66]

**Fig. 5:**
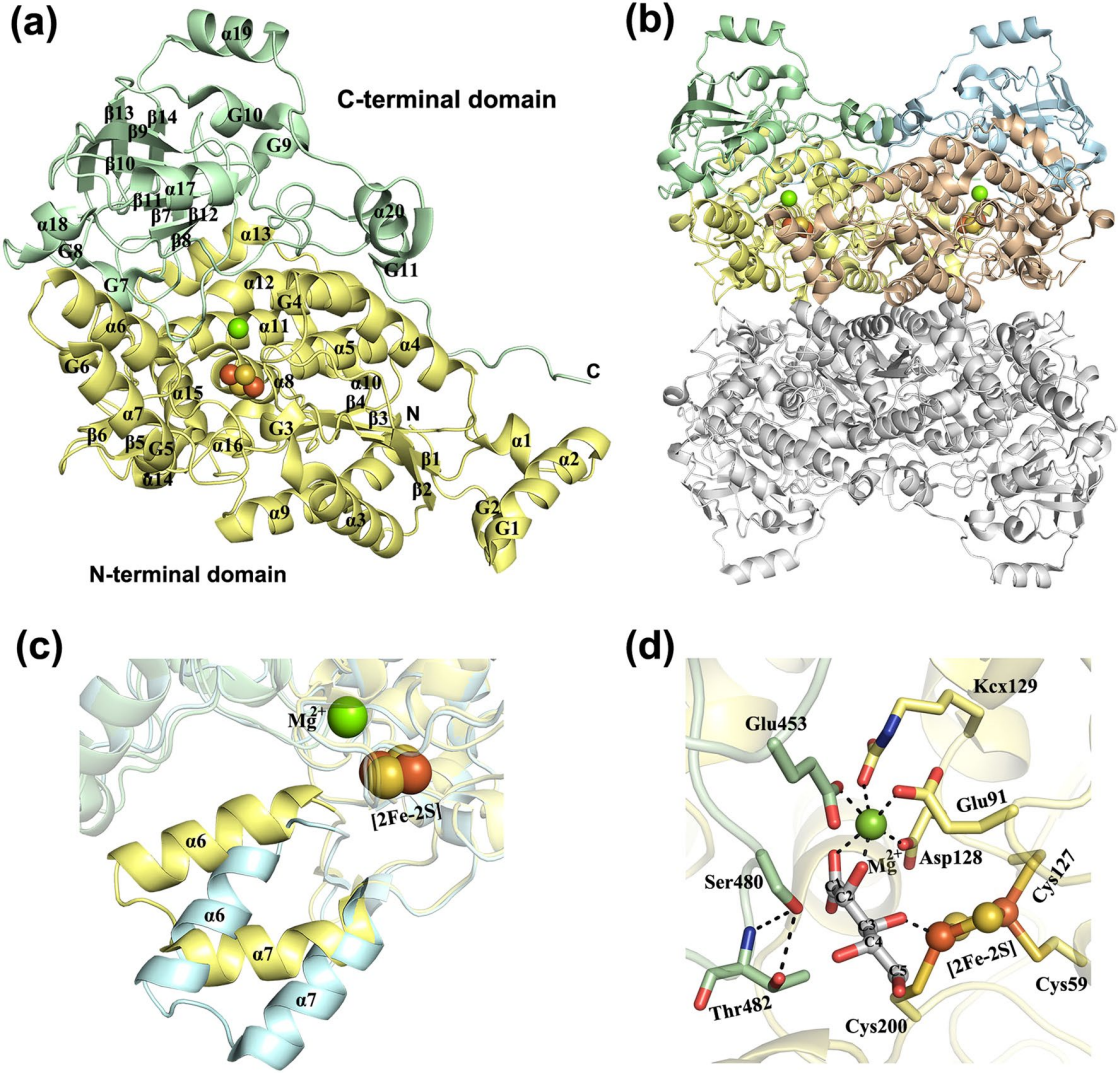
Crystal structure of *Rl* ADHT (PDB: 5J84). **a** Overall fold is represented by a ribbon diagram. The N-terminal domain is shown in yellow, and the C-terminal domain is shown in green. The Mg^2+^ ion is represented by a green sphere and the [2Fe–2S] cluster by orange–yellow spheres. **b** Overall structure of the homo-tetramer. **c** Conformational shift of the N-terminal helix − loop − helix region. The closed form (PDB: 5J84) is shown in yellow and the open form (PDB: 5J85) in cyan. **d** Active site of *Rl* ADHT (PDB: 5J84) with a docked l-Arabinonate

The overall structure of *Rl* ADHT, as well as all other IlvD/EDD enzymes, consists of two domains, the N-terminal αβα-sandwich domain and the C-terminal β-barrel domain (Fig. 5a). The core of the N-terminal domain is formed by a four-stranded parallel β-sheet flanked by α-helices, which is then further surrounded by additional secondary structures. The C-terminal domain consists of eight mixed β-strands. IlvD/EDD proteins exist as either homodimers or homotetramers, but the tetrameric structure can be also described as a dimer of dimers (Fig. 5b). Dimerization has a significant role in formation of the active site, which lies in the cavity between the two domains and at the interface of the monomeric units of the dimer. Dimerization restricts free access to the active site and protects the iron–sulfur cluster from oxidative damage, but transient access is achieved by a conformational shift of the N-terminal helix − loop − helix region as shown in the structure of the *Rl* ADHT S480A mutant [58] (Fig. 5c).

Each monomer of the aldopentonate dehydratases contains an iron–sulfur [2Fe–2S] cluster and an Mg^2+^ ion in the active site. In *Rl* ADHT, a planar [2Fe–2S] cluster is bound to the N-terminal domain by three cysteine residues (Cys59, Cys127, and Cys200). All the published structures of IlvD/EDD enzymes show that [2Fe–2S] cluster is coordinated by three cysteines, and the fourth site is thought to be filled either by water or substrate. Mg^2+^ is octahedrally coordinated by a conserved Asp, two Glu and ɛ-carboxy-Lys residues, along with two water molecules. A Mg^2+^ ion most likely coordinates the sugar acid at its C1 carboxylate group to the active site. Based on solved crystal structures and site-directed mutagenesis studies, the reaction mechanism of aldopentonate dehydratases is suggested to begin by the abstraction of a proton from the C2 atom of aldopentonate by the alkoxide form of the conserved serine side chain (Ser480 in *Rl* ADHT and Ser490 in *Cc* XyDHT), which acts as a Lewis base [58]. A three-coordinated Fe atom acts as a Lewis acid and accepts an electron pair from the leaving hydroxyl group on the C3 of the substrate.

### Enolase superfamily

Although no crystal structure of aldopentonate dehydratase belonging to the enolase superfamily is currently described, several crystal structures of aldohexonate and deoxyhexonate dehydratases—such as d-glucarate dehydratases [71, 72], l-rhamnonate dehydratase [73], l-fuconate dehydratase [74], galactarate dehydratase [75] and d-mannonate dehydratase [76]—are known. Based on variations in which amino-acid residues participate in catalysis, the enolase superfamily can be divided into the following seven subgroups: enolase, muconate lactonizing enzyme, mandelate racemase, d-glucarate dehydratase, d-mannonate dehydratase, β-methylaspartate ammonia lyase, and galactarate dehydratase [77]. Archeal aldopentonate dehydratases, such as from *H. volcanii*, and 2 dehydratases from *S. solfataricus,* belong to the mandelate racemase/muconate lactonizing enzyme family of the enolase superfamily along with d-gluconate/d-galactonate dehydratase from *S. solfataricus* (*Ss* GADHT) of the modified promiscuous ED pathway. *Ss* GADHT can convert d-gluconate and d-galactonate, but is unable to convert aldopentonates. The crystal structure of *Ss* GADHT is also currently unknown. Dehydratase from *H. volcanii* is reported to catalyze d-xylonate and d-gluconate [69], while Sso3124 *S. solfataricus* dehydratase [30] participates in d-arabinose degradation, and Sso2665 *S. solfataricus* dehydratase participates in degradation of d-xylonate and l-arabinonate [37].

Based on sequence comparison, it can be predicted that archaeal aldopentonate dehydratases contain a typical enolase fold with two domains: an N-terminal α + β capping domain and a C-terminal modified TIM-barrel domain, known as a (β/α)_7_β-barrel. Both *H. volcanii* and Sso3124 *S. solfataricus* dehydratases are characterized as homo-octamers, whereas Sso2665 *S. solfataricus* dehydratase is active as a tetramer [30, 69]. The N-terminal domain is primarily responsible for determining substrate specificity and the C-terminal domain is responsible for acid/base chemistry. The active site is located at the interface between the domains and contains a Mg^2+^ ion. The Mg^2+^ ion is suggested to play an essential role in the reaction mechanism as it stabilizes an enolate anion intermediate generated by the abstraction of a carboxylate α-proton by an active site base as the active site acid usually directs intermediates toward the product [77].

### 2*-Dehydro-3-deoxy-aldopentonate dehydratases*

In the penultimate reaction step of the Weimberg pathway, 2-dehydro-3-deoxy-aldopentonate is converted to 2,5-dioxopentanoate (often called α-ketoglutaric semialdehyde) by 2-dehydro-3-deoxy-aldopentonate dehydratase. The l-form intermediate of the pathway, 2-dehydro-3-deoxy-l-aldopentonate, is dehydrated by 2-dehydro-3-deoxy-l-aldopentonate dehydratase (EC 4.2.1.43), a.k.a. 2-dehydro-3-deoxy-l-arabinonate dehydratase, or 2-keto-3-deoxy-l-arabinonate dehydratase. The d-form intermediate, 2-dehydro-3-deoxy-d-aldopentonate, is converted by 2-dehydro-3-deoxy-d-aldopentonate dehydratase (EC 4.2.1.141), a.k.a. 2-dehydro-3-deoxy-d-arabinonate dehydratase, or 2-keto-3-deoxy-d-arabinonate dehydratase. This enzyme is also sometimes called 2-dehydro-3-deoxy-d-xylonate dehydratase due to its role in d-xylose degradation. However, the penultimate intermediate of the Weimberg pathway is the same for all d-aldopentoses, because only one stereocenter is left from the pentose sugar.

Interestingly, enzymes catalyzing different stereoisomers have remarkably different three-dimensional structures. Table 4 shows the available crystal structures of 2-dehydro-3-deoxy-aldopentonate dehydratases. The crystal structure of *S. solfataricus* 2-dehydro-3-deoxy-d-aldopentonate dehydratase (hereafter referred to as *Ss* DPDHT) has been solved with bound magnesium (PDB code 3BQB) and with calcium ions (PDB code 2Q1C), and also complexed with a substrate analogue 2-oxobutyrate (PDB code 2Q1A) and a product 2,5-dioxopentanoate (PDB code 2QID) [78]. *Ss* DPDHT belongs to the metal-dependent fumarylacetoacetate hydrolase (FAH) protein family. By contrast, 2-dehydro-3-deoxy-L-aldopentonate dehydratase from *A. brasilense* (hereafter referred as *Ab* LPDHT) belongs to dihydrodipicolinate synthase/N-acetylneuraminate lyase (DHDPS/NAL) protein family. In 2008, the crystal structure of *Ab* LPDHT was determined, both in its uncomplexed form (PDB code 3FKK) [79] and complexed with pyruvate (PDB code 3FKR) [80]. Recently, crystal structures of *Ab* LPDHT have been determined without a ligand (PDB code 7C0C) and complexed with two different substrate analogues: β-hydroxypyruvate (PDB code 7C0D) and 2-oxobutyrate (PDB code 7C0E) [81].

**Table 4:** Available crystal structures of 2-dehydro-3-deoxy-aldopentonate dehydratases

Enzyme (Short name in this article) EC number	PDB code	Organism	Resolution (Å)	Ligands	Quaternary structure	Refs.
FAH superfamily						
2-Dehydro-3-deoxy-d-aldopentonate dehydratase (Ss DPDHT) EC 4.2.1.43	2Q18 2Q19 2Q1A 2Q1C 2Q1D 3BQB	*Sulfolobus solfataricus*	2.10 3.00 2.50 2.80 2.70 2.70	– – Mg^2+^, 2-oxobutyrate Ca^2+^, 2-oxobutyrate Mg^2+^, 2,5-dioxopentanoate Mg^2+^	Tetramer	[78]
DHDPS/NAL superfamily						
2-Dehydro-3-deoxy-l-aldopentonate dehydratase (*Ab* LPDHT) EC 4.2.1.43	3FKK 3FKR 7C0C 7C0D 7C0E	*Azospirillum brasilense*	2.10 1.80 1.90 1.60 2.20	– Pyruvate – Hydroxypyruvate 2-oxobutyrate	Tetramer	[79–81]

### FAH superfamily

**Fig. 6:**
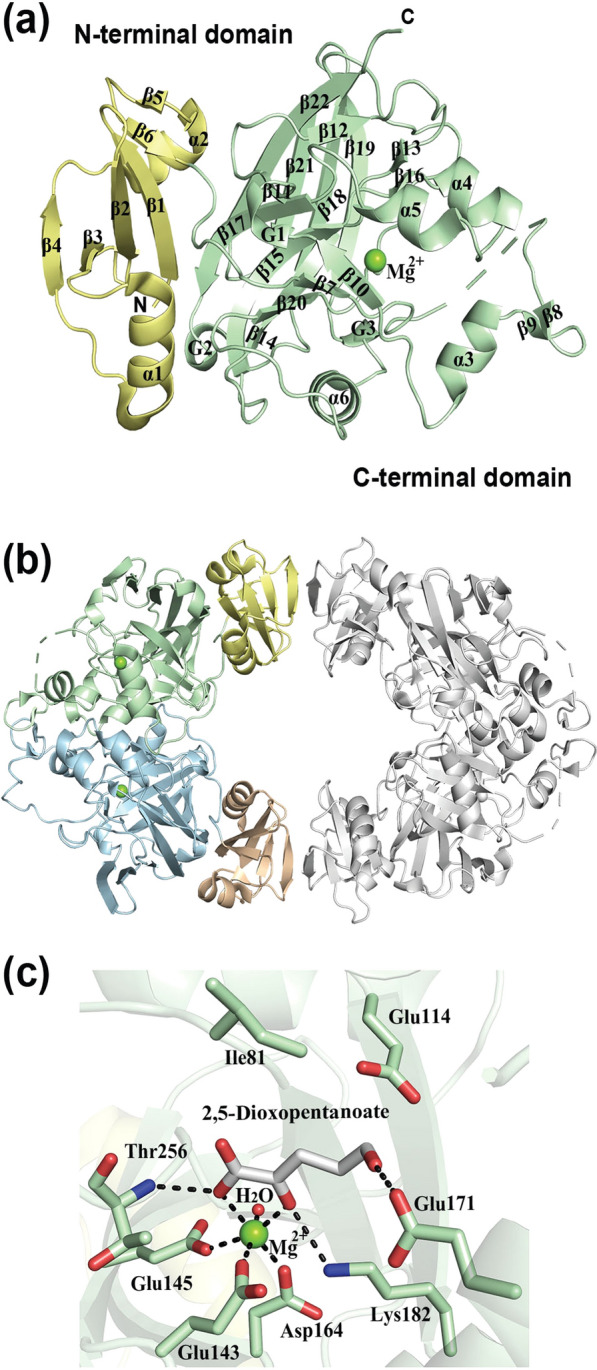
Crystal structure of *Ss* DPDHT (PDB: 3BQB). **a** Overall fold is represented by a ribbon diagram. The N- and C-terminal domains are shown in green and yellow, respectively. Mg.^2+^ ion shown as green sphere. **b** Overall structure of the homo-tetramer. **c** Crystal structure of *Ss* DPDHT complexed with a 2,5-dioxopentanoate product (PDB: 2Q1D)

The FAH superfamily is named after fumarylacetoacetate hydrolase, which was the first solved protein structure in the family [82]. The family contains a diverse set of enzymes that are essential for degrading complex carbon sources in various metabolic pathways in both prokaryotes and eukaryotes. Despite the enzymatic diversity, all FAH superfamily members contain a catalytic FAH-domain. *Ss* DPDHT has two domains: an N-terminal domain with a four-stranded antiparallel β-sheet flanked by two α-helices on either side, and a catalytic C-terminal FAH-domain with a mixed β-sandwich roll fold (Fig. 6a). The quaternary structure of *Ss* DPDHT is an oval-ring shaped homotetramer, composed of a dimer of dimers (Fig. 6b).

The active site pocket contains a catalytic Mg^2+^ ion that is coordinated by three conserved acidic residues (Glu143, Glu145, and Glu164 in *Ss* DPDHT). The metal ion is hexacoordinated by these three acidic residues together with one water molecule and two oxygen atoms of the ligand (Fig. 6c). The enzymatic mechanism for water elimination from 2-dehydro-3-deoxy-d-aldopentonate involves the catalytic metal ion and a catalytic dyad, Glu/Lys. Two putative reaction mechanisms for dehydration of 2-dehydro-3-deoxy-d-arabinonate have been proposed [78], which are differentiated by initial proton abstraction from either C3 or C5. In both mechanisms, it is suggested that the Glu114 subtracts a proton and Lys182 adds a proton to the C4 hydroxyl group to cause water elimination. The Mg^2+^ ion holds the substrate in place and the bidentate binding of the substrate might increase the acidity of the C3 proton.

### DHDPS/NAL family

**Fig. 7:**
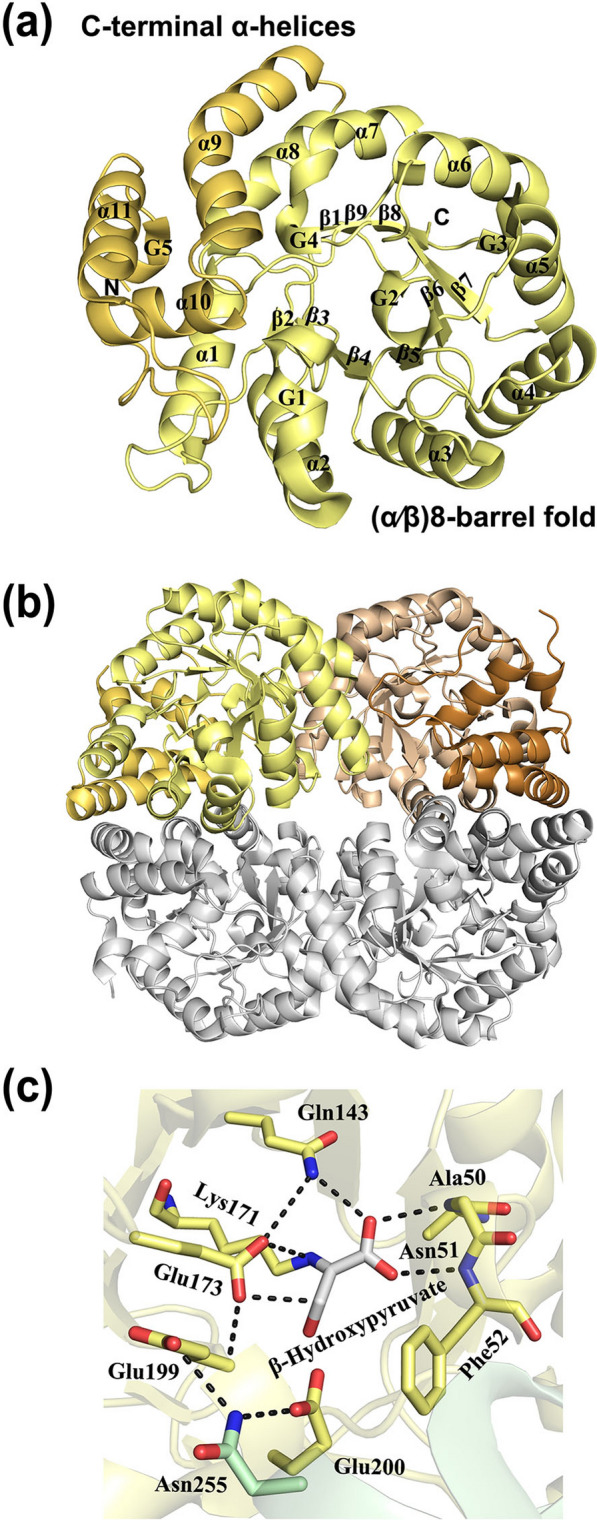
Crystal structure of the monomer of *Ab* LPDHT (PDB: 7C0C). **a** Overall fold is represented by a ribbon diagram. The (α/β)8-barrel fold is shown in yellow; C-terminal α-helices are shown in light orange. **b** Overall structure of the homo-tetramer. **c** Active site of *Ab* LPDHT complexed with the substrate analogue β-hydroxypyruvate (PDB: 7C0D)

The DHDPS/NAL protein family is named after the first two solved archetypal structures: dihydrodipicolinate synthase (DHDPS) [83] and N-acetylneuraminate lyase (NAL) [84]. This is a large family of enzymes, such as 5-keto-4-deoxy-glucarate dehydratases and d-2-keto-deoxy-gluconate aldolases. DHDPS/NAL proteins have a common (α/β)_8_-barrel fold composed of a (β)_8_-barrel surrounded by eight α-helices, where the active site is at the C-terminal end of a central β-barrel. In addition, the structure contains three C-terminal α-helices (Fig. 7a). DHDPS/NAL proteins are tetramers composed of dimers of dimers (Fig. 7b). The wide interface area between the monomers suggests that the tetrameric structure is highly stable in solution.

The active site of the DHDPS/NAL enzyme contains a conserved lysine residue that forms a Schiff-base intermediate with the C2 carbon of an α-keto acid substrate, usually performing cleavage of C–C or C=C bonds typical of type I aldolases. The 2-dehydro-3-deoxy-l-aldopentonate dehydratase lacks the aldol cleavage functionality and performs only the dehydration reaction. In *Ab* LPDHT, Lys171 is located at the center of the barrel on β6 and mutation of conserved lysine to alanine completely inactivates the enzyme, suggesting Schiff-base formation also occurs with 2-dehydro-3-deoxy-l-arabinonate dehydratases [81]. Interestingly, 2-dehydro-3-deoxy-l-aldopentonate dehydratase seems to have a generally poor phylogenetic relationship with other DHDPS/NAL enzymes. It is thought that 2-dehydro-3-deoxy-l-aldopentonate dehydratase evolved from a common aldolase ancestor with a tyrosine residue having been replaced by Gln143 in Ab LPDHT. In addition, the substrate-binding motif in *Ab* LPDHT, which is significantly divergent and involved in acid/base catalysis in type 1 aldolases, has been replaced.

Based on crystallographic and mutagenesis studies, a reaction mechanism for 2-dehydro-3-deoxy-l-aldopentonate dehydratases has been proposed [81], where Glu173 and Glu200 acts as catalytic Brønsted bases for the C3 and C5 protons, respectively, of the Schiff-base intermediate. The C3 atom of hydroxypyruvate (and also the C3 atom from 2-oxobuturate) is about 3 Å away from the carboxylate group of Glu173 (Fig. 7c). Glu173 also interacts with a side-chain of Gln143 and the main-chain of Glu199. Notably, Glu173 is not conserved among any other DHDPD/NAL enzymes. In aldolases, the tyrosine residue typically acts as a Brønsted base catalyst, resulting in cleavage of the substrate.

## Ketoglutarate-semialdehyde dehydrogenase

The last step of the Weimberg pathway is the conversion of 2,5-dioxopentanoate to α-ketoglutarate via ketoglutarate-semialdehyde dehydrogenase (E.C. 1.2.1.26). Ketoglutarate-semialdehyde dehydrogenase belongs to the aldehyde dehydrogenase (ALDH) family, which contains a variety of NAD(P)-dependent enzymes that catalyse the oxidation of aliphatic and/or aromatic aldehydes to their corresponding acids. ALDH proteins are found in all kingdoms and have multiple functions in cellular metabolism and defense systems [85]. In 1997, Liu et al. [86]. were the first to solve the structure of aldehyde dehydrogenase from *Rattus norvegicus.*

At present, structural information for ketoglutarate-semialdehyde dehydrogenases is very limited (Table 5) and only the crystal structure of ketoglutarate-semialdehyde dehydrogenase from *A. brasiliensis* (hereafter referred to as *Ab* KGSADH) has been solved with and without a cofactor (PDB codes 5X5T and 5X5U) [87].

**Table 5:** Available crystal structures of ketoglutarate-semialdehyde dehydrogenases

Enzyme (Short name in this article) EC number	PDB code	Organism	Resolution (Å)	Ligands	Quaternary structure	Refs.
Ketoglutarate-semialdehyde dehydrogenase (*Ab* KGSADH) E.C. 1.2.1.26	5X5T 5X5U	*Azospirillum brasilense*	2.25 2.30	– NAD	Tetramer	[87]

**Fig. 8:**
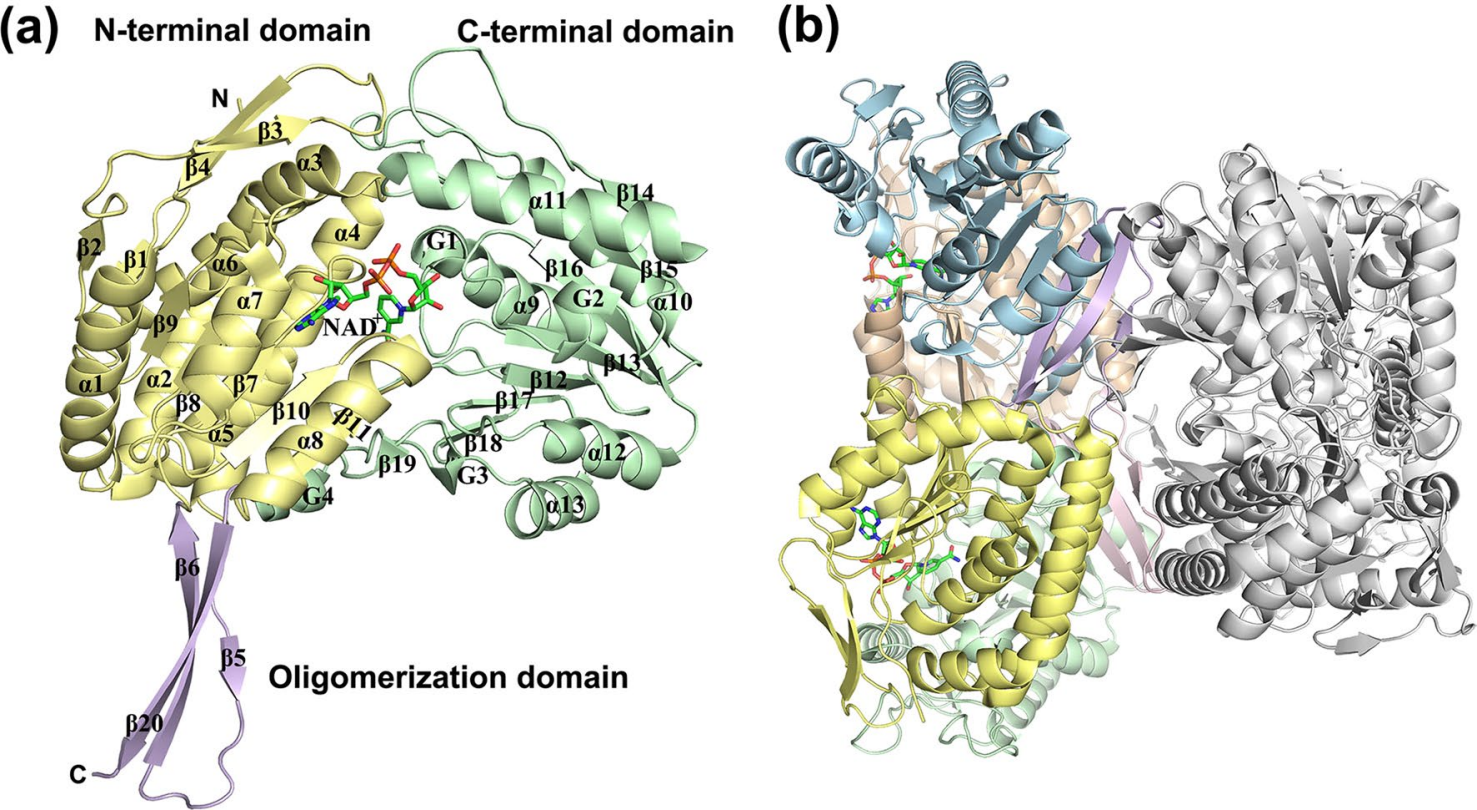
Crystal structure of the monomer of the ketoglutarate-semialdehyde dehydrogenase *Ab* KGSADH from *Azospirillum brasilense* (PDB: 5X5U). **a** Overall fold is represented by a ribbon diagram. The N-terminal domain, C-terminal domain and C-terminal oligomerization domains are shown in yellow, green, and purple, respectively. **b** Overall quaternary structure is a homotetramer

The *Ab* KGSADH has a three-domain structure typical of ALDH proteins: 1) an N-terminal domain (Met1–Arg123 and Val145–Leu254), 2) an oligomerization domain (Val124–Pro144 and Tyr470–Val481), and 3) a C-terminal domain (Gly255–Pro469) (Fig. 8a). The N-terminal domain is responsible for nucleotide binding and is composed of seven α-helices (α1–α7) and nine β-strands (β1–β4 and β7–β11), the core of which has a five-stranded Rossmann-like fold (β7–β11). The C-terminal domain has a large β-sheet containing seven β-strands (β12–β18) surrounded by six α-helices (α8–α13) and two 3_10_-helices (G1, G2). In addition, two 3_10_-helices (G3, G4) and one short β-strand (β19) exist at the C-terminus of this domain. The oligomerization domain is composed of a three-stranded antiparallel β-sheet (β5, β6, and β20) that protrudes from the N-terminal domain. In the dimer, the last β-strand of the oligomerization domain interacts with the last β-strand of the C-terminal domain of another monomeric subunit. The quaternary structure of Ab KGSADH is a homotetramer, a dimer of dimers (Fig. 8b), which is a highly conserved architecture among ALDH proteins.

*Ab* KGSADH prefers to utilize NAD as a cofactor [53]. The NAD binding pocket lies in a space between the N- and C-terminal domains. In NAD-dependent ALDHs, the ribose-ring binding site of an adenosine nucleotide is not large enough to accommodate the phosphorylated form of the ring, which accounts for the preference of NAD over NADP. Many NADP-dependent ALDHs have a serine residue instead of the Glu181 residue observed in *Ab* KGSADH.

The active site tunnel exists in the inter-domain space, where two highly conserved catalytic Cys and Glu residues are found at the bottom of the pocket. In *Ab* KGSADH, the catalytic residues were concluded from site-directed mutagenesis experiments to be Cys287 and Glu253 [87]. During catalysis, the Cys residue first forms a tetrahedral intermediate with the carbonyl carbon of the aldehyde group, and the hydride ion is then transferred to NAD. The Glu residue acts as a general base and abstracts a proton from a water molecule, which then attacks the carbonyl carbon to again form a tetrahedral intermediate. The acidic product dissociates and the enzyme is then ready for a new cycle of catalysis [88].

### 2*-Dehydro-3-deoxy-aldopentonate aldolase*

In the Dahms pathway, the last step is catalyzed by 2-dehydro-3-deoxy-aldopentonate aldolase (a.k.a. 2-keto-3-deoxy-aldopentonate aldolase), which cleaves 2-dehydro-3-deoxy-aldopentonate into pyruvate and glycolaldehyde. Currently, no crystal structures of aldolases with strict specificity to 2-dehydro-3-deoxy-aldopentonate are described. However, several Entner–Doudoroff pathway-associated 2-dehydro-3-deoxy-gluconate aldolases, such as from *S. solfataricus* (hereafter referred to as *Ss* KDGA), *S. acidocaldarius* (hereafter referred to as *Sa* KDGA), *S. tokodaii* [37, 89]*,* and *E. coli* (hereafter referred to as *Ec* KDGA) [17], have been reported to have some activity for aldopentonates (Table 6). Interestingly, these enzymes have been reported to have catalytic activity on both C4 epimers of hexose and pentose sugars as they cleave both 2-dehydro-3-deoxy-gluconate and 2-dehydro-3-deoxy-galactonate as well as both 2-dehydro-3-deoxy-l-aldopentonate and 2-dehydro-3-deoxy-d-aldopentonate [90]. Several crystal structures of *Ss* KDGA, *Sa* KDGA and *Ec* KDGA have been solved with and without ligands, such as pyruvate, 2-dehydro-3-deoxy-gluconate or 2-dehydro-3-deoxy-galactonate [89, 91–93].

**Table 6:** Available crystal structures of 2-dehydro-3-deoxy-d-gluconate aldolases with reported 2-dehydro-3-deoxy-aldopentonate activity

Enzyme (Short name in this article) EC number	PDB code	Organism	Resolution (Å)	Ligands	Quaternary structure	Refs.
2-Dehydro-3-deoxy-d-gluconate aldolase (*Ss* KDGA) EC 4.1.2.51	1W37 1W3I 1W3N 1W3T 6G3Z	*Saccharolobus solfataricus*	2.00 1.70 2.10 2.10 2.35	– Pyruvate 2-keto-3-deoxy-d-gluconate 2-keto-3-deoxy-d-galactonate, d-glyceraldehyde, pyruvate 2-keto-3-deoxy-6-phosphogluconate	Tetramer	[91, 94]
2-Dehydro-3-deoxy-d-gluconate aldolase (*Sa* KDGA) EC 4.1.2.51	2NUW 2NUX 2NUY	*Sulfolobus acidocaldarius*	1.80 2.50 2.50	– – Pyruvate	Tetramer	[89]
2-Dehydro-3-deoxy-d-gluconate aldolase (*Ec* KDGA) EC 4.1.2.51	2V8Z 2V9D 3N2X 3NEV 4OE7 4ONV 4PTN 4U4M	*Escherichia coli*	2.20 2.15 2.20 2.19 1.99 2.57 1.99 3.09	– – Pyruvate 2-keto-3-deoxy-d-galactonate 4-hydroxy-2,5-DOP, pyruvate, ethanedial, Mg^2+^ 2-keto-3-deoxy-d-gluconate l-glyceraldehyde, Mg^2+^ pyruvate	Tetramer	[92, 93, 95–98]

The 2-dehydro-3-deoxy-aldopentonate aldolases belong to the DHDPS/NAL protein family along with the 2-dehydro-3-deoxy-l-aldopentonate dehydratases discussed earlier. However, compared with 2-dehydro-3-deoxy-l-aldopentonate dehydratase, 2-dehydro-3-deoxy-aldopentonate aldolase is expected to be a far more conventional C–C bond cleaving member of the DHDPS/NAL protein family as its active site contains the conserved catalytic residues lysine and tyrosine, and conserved substrate-recognizing residues (GXXG motif). Lysine is responsible for formation of a Schiff-base intermediate, and tyrosine is responsible for shuttling protons. Mutagenesis studies have shown that the catalytic triad of Tyr, Thr (or Ser) and Tyr residues act as a shuttle to transfer protons to and from active site that is needed for formation of a Schiff´s base and subsequent aldol condensation/cleavage [91]. In the 2-dehydro-3-deoxy-d-gluconate aldolase *Ec* KDGA, the triad is formed by Tyr145 above the Schiff´s base-forming Lys174, Ser56 from the GXXG motif, and Tyr119 from the adjacent monomer in the dimer [93].

**Fig. 9:**
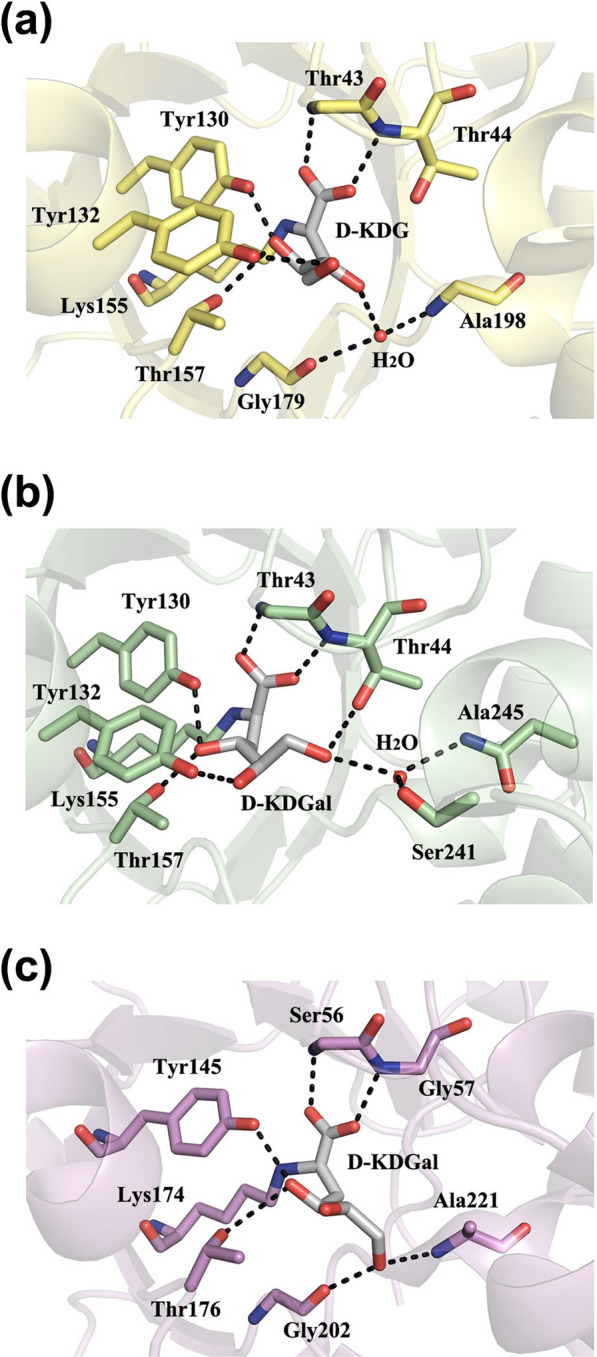
Active site of KDGA enzymes: **a** *Ss* KDGA complex with D-KDG (PDB: 1W3N); **b** *Ss* KDGA complex with D-KDGal (PDB: 1W3T); and **c** *Ec* KDGA complex with D-KDGal (PDB: 3NEV)

2-Dehydro-3-deoxy-d-gluconate aldolases catalyse cleavage of 2-dehydro-3-deoxy-d-gluconate to pyruvate and glyceraldehyde, but they also possess a catalytic promiscuity that enables them to catalyse 2-dehydro-3-deoxy-d-galactose as well. The Schiff´s base complexes of *Ss* GDGA with C4 epimers of 2-dehydro-3-deoxy-d-glucose (D-KDG) and 2-dehydro-3-deoxy-d-galactose (D-KDGal) show that the active site of the enzyme is rigid, but the sugar substrates have conformational flexibility (Fig. 9a, b). The O4-hydroxyls of both substrates are in positions to interact with Tyr130, supporting the role of this tyrosine in proton abstraction. An alternative hydrogen-bonding network allows recognition of O5- and O6-hydroxyls even when they are in different orientations, which explains the substrate promiscuity of *Ss* GDGA [91]. The *Ec* KDGA–D-KDGal complex shows that its interactions with substrates are similar to those observed in the *Ss* KDGA–D-KDGal complex, but the O6–hydroxyl interacts directly with the backbone of Gly210 and Ala221 (Fig. 9c).

## Future prospects

**Fig. 10:**
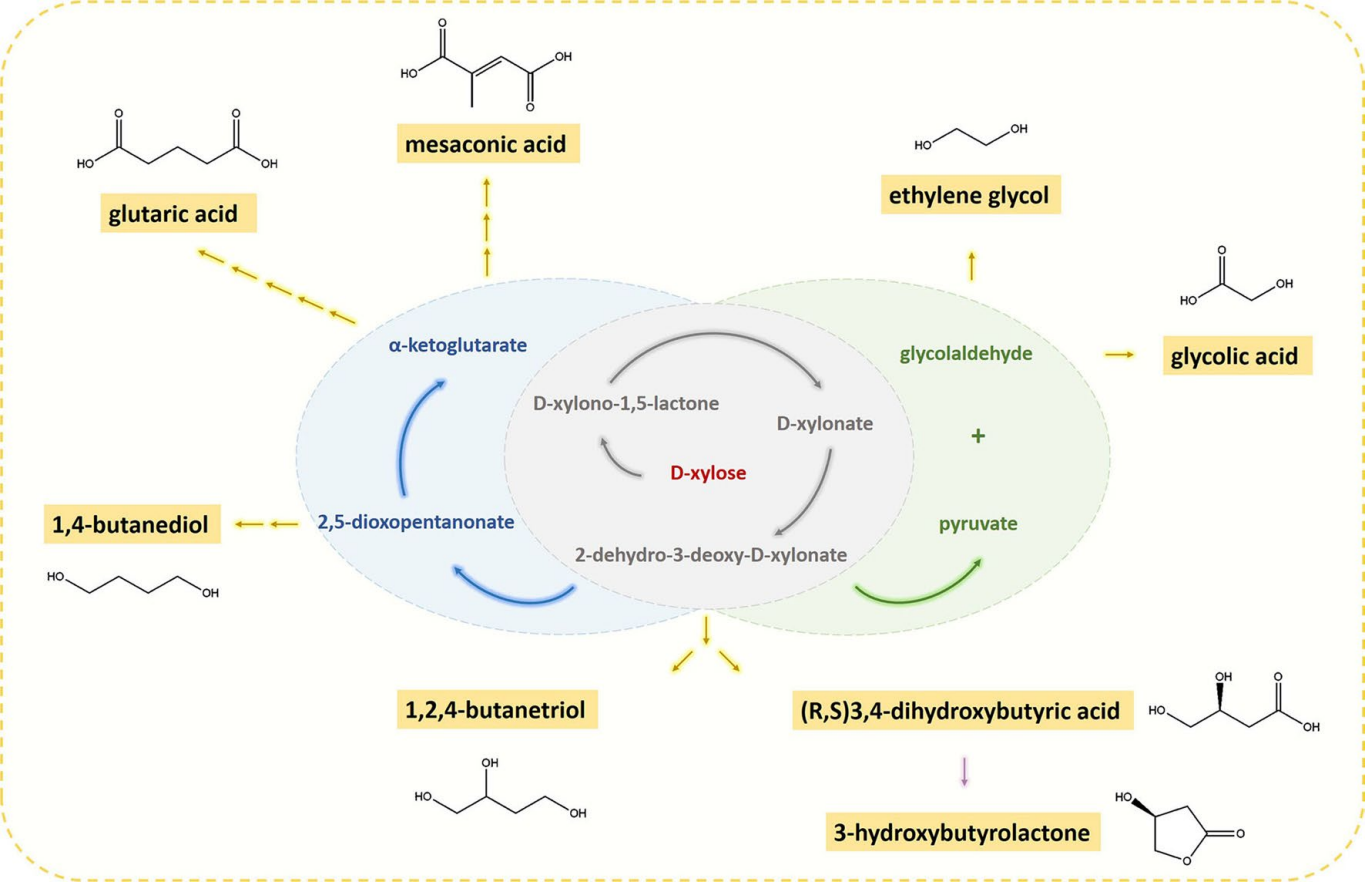
Added-value chemicals that can be produced from the intermediates or products of Weimberg/Dahms pathway. In the center of the scheme, the gray circle contains the common steps for Weimberg and Dahms pathways, the blue circle have the subsequent two steps in Weimberg pathway, and the green circle shows the last step of Dahms pathway. The added-value chemicals that can be derived from the pathways are shown. The yellow arrows correspond the enzymatic reaction and the purple arrow is the non-enzymatic reaction at acidic condition

The oxidative Weimberg and Dahms pathways provide five- and four-step enzymatic reactions, respectively, for converting pentose sugars to key glycolytic intermediates, such as α-ketoglutarate in the Weimberg pathway, and pyruvate and glycolaldehyde in the Dahms pathway, thus offering sustainable bioconversion of lignocellulose-derived aldopentoses to added-value products (Fig. 10). Weimberg/Dahms pathways are also independent of other carbohydrate assimilation pathways, which simplifies metabolic engineering efforts [99]. Oxidative aldopentose pathway enzymes have been utilized for production of d-xylonic acid or l-arabinoic acid from biomass-derived aldopentoses, either in *E.coli* or various yeasts [31, 44, 100]. Besides these pentonic acids, microbial synthesis of 1,2,4-butanetriol by the oxidative aldopentose pathway has been demonstrated [101]. A similar synthetic route, with a different last step reaction, of 3,4-dihydroxybutyric acid has also been established in *E. coli* [102]. At acidic condition, 3,4-dihydroxybutyric acid can be cyclized to 3-hydroxybutyrolactone (γHBL). Liu et al. [17, 103] have further reported biosynthesis of ethylene glycol from d-xylose by the Dahms pathway in *E.coli*, while Salusjärvi et al. [61] produced ethylene glycol and glycolic acid in *S. cerevisiae* yeast. This pathway of glycolic acid synthesis has also been used for PLGA copolymer (of glycolic acid and lactic acid) production in *E. coli* [104, 105]*.* In addition, Tai et al. [3] have applied partial Weimberg pathway for producing 1,4-butanediol in *E. coli*. The Weimberg pathway has been also utilized to yield mesaconic acid [106] as well as the glutaric acid via the α-ketoglutarate [107] in *E. coli*.

Metabolic engineering efforts have shown their potential in the production of valuable bioproducts from aldopentoses. However, incomplete biochemical and structural knowledge of the enzymes in these metabolic systems is still hampering these efforts. For example, it is evident that one of the bottlenecks in these oxidative pathways is the Fe–S cluster-containing aldopentonate dehydratase enzyme, which should be optimized in terms of expression and catalytic efficiency [49, 61, 99]. Three-dimensional enzyme structures and biochemical data are needed to provide a better understanding of structure–function relationships of the enzymes, including catalytic mechanisms, as well as to help protein and metabolic engineering efforts.

Protein engineering, including future novel artificial enzymes, provides a tremendous toolbox to broaden the potential applications of synthetic biochemical routes. The oxidative non-phosphorylative pathways of pentose catabolism have not yet been fully exploited, and the involving enzymes have not yet been subjected to protein engineering efforts. Particularly, the improvement of bottleneck enzyme, the aldopentonate dehydratase, will be beneficial to solve the drawbacks of utilizing these pathways in biosynthesis of chemicals from pentose sugars [99]. On the other hand, the optimization of some other related enzymes has been reported. Naturally existing diol dehydratase has been designed by rational protein engineering to have catalytic activity toward the artificial substrate 1,2,4-butanetriol, which led to enhanced production of 1,4-butanediol from xylose [108]. In addition to rational protein design, more random approaches have been used to modify the enzymes. Recently, dihydroxyacid dehydratase from *S. solfataricus* was successfully engineered to have tenfold higher activity for conversion of glycerate dehydration to pyruvate by iterative saturation mutagenesis [109].

The main aim of this review has been to provide a summary of the existing three-dimensional structures of enzymes involved in oxidative non-phosphorylative catabolic pathways for aldopentoses, as well as to highlight some missing links, including complex structures of enzymes, that may restrict understanding of enzymatic reactions. We believe that our structural review of Weimberg/Dahms pathway enzymes will be of value for researchers attempting to engineer more efficient pathways for producing biobased compounds from xylose and arabinose. Engineered enzyme pathways could be used in vivo or in vitro, or in hybrid systems. Ultimately, we hope that an increased range of biobased products can be developed to help reduce our dependence on oil by replacement with renewable energy sources.

## Data Availability

Not applicable.

## Abbreviations

AAOR: Aldose-aldose oxidoreductase
ADH: Arabinose dehydrogenase
ADHT: Arabinonate dehydratase
ALDH: Aldehyde dehydrogenase
DHDPS: Dihydrodipicolinate synthase
DPDHT: 2-Dehydro-3-deoxy-d-aldopentonate dehydratase
ED: Entner–Doudoroff
EDD: Entner–Doudoroff dehydratase
FAH: Fumarylacetoacetate hydrolase
GADHT: Gluconate/galactonate dehydratase
GDH: Glucose dehydrogenase
IlvD: Isoleucine/leucine/valine dehydrate
KDG: 2-Keto-3-deoxy-glucose
KDGA: 2-Keto-3-deoxy-gluconate aldolases
KDGal: 2-Keto-3-deoxy-galactose
KGSADH: Ketoglutarate-semialdehyde dehydrogenase
LDR: Long-chain dehydrogenase/reductase
LPDHT: 2-Dehydro-3-deoxy-l-aldopentonate dehydratase
MDR: Medium-chain dehydrogenase/reductase
NAD: Nicotinamide adenine dinucleotide
NADP: Nicotinamide adenine dinucleotide phosphate
NAL: N-acetylneuraminate lyase
PDB: Protein data bank
PLGA: Poly lactic-*co*-glycolic acid
SDR: Short-chain dehydrogenase/reductase
SMP30: Senescence marker protein 30
XDHT: Xylonate dehydratase
